# Women Are More Infected and Seek Care Faster but Are Less Severely Ill: Gender Gaps in COVID-19 Morbidity and Mortality during Two Years of a Pandemic in Israel

**DOI:** 10.3390/healthcare10122355

**Published:** 2022-11-23

**Authors:** Arielle Kaim, Shani Ben Shetrit, Mor Saban

**Affiliations:** 1 Department of Emergency and Disaster Management, Faculty of Medicine, School of Public Health, Sackler Tel Aviv University, Tel Aviv 6139001, Israel; 2 Israel National Center for Trauma & Emergency Medicine Research, The Gertner Institute for Epidemiology and Health Policy Research, Sheba Medical Center, Ramat Gan 5266202, Israel; 3 The Buchmann Faculty of Law, Tel Aiv University, Tel Aviv 6139001, Israel; 4 Health Technology Assessment and Policy Unit, The Gertner Institute for Epidemiology & Health Policy Research, Sheba Medical Center, Ramat Gan 5266202, Israel; 5 Nursing Department, Sackler Faculty of Medicine, School of Health Professions, Tel-Aviv University, Tel Aviv 6139001, Israel

**Keywords:** COVID-19, pandemic, gender, prevention, disparities

## Abstract

In the context of COVID-19 outcomes, global data have deduced a gender bias towards severe disease among males. The aim is to compare morbidity and mortality during two years of the COVID-19 pandemic in female and male patients with COVID-19, as well as to assess length of stay, time of health-seeking behavior after positive diagnosis, and vaccination differences. A retrospective-archive study was conducted in Israel from 1 March 2020 to 1 March 2022 (two consecutive years). Data were obtained from the Israeli Ministry of Health’s (MOH) open COVID-19 database. The findings indicate female infections are 1.12 times more likely, across almost all age groups, apart from the youngest (0–19) age groups. Despite this, the relative risk of severe illness, intubation and mortality is higher among men. In addition, our findings indicate that the mean number of days taken by unvaccinated men from positive diagnosis to hospital admission was greater than among unvaccinated women among the deceased population. The findings of this study reveal lessons learned from the COVID-19 global pandemic. Specifically, the study shows how human biological sex may have played a role in COVID-19 transmission, illness, and death in Israel. The conclusions of this study indicate that targeted approaches, which take into consideration sex and gender and the intersecting factors are necessary to engage in the fight against COVID-19 and ensure the most effective and equitable pandemic response.

## 1. Introduction

Over two years into the COVID-19 pandemic, the virus continues to be an ongoing global threat, with over 635 million diagnosed cases and 6.6 million deaths worldwide as of 15 November 2022 [1]. At the initial stages of the pandemic, emphasis was initially placed on elderly or among those with preexisting health conditions as being at high-risk of contracting the virus or death; however, human biological sex has been documented to play a central role in heterogeneous infectious disease pathogenesis [2,3,4].

These clinical findings are consistent with previous outbreaks of highly pathogenic coronaviruses such as severe acute respiratory syndrome coronavirus (SARS-CoV-1) and the Middle East respiratory syndrome coronavirus (MERS-CoV), where men were more likely to have been infected and have worse outcomes [5,6].

In the context of COVID-19 outcomes, similarly, global data have deduced a gender bias towards severe disease among males [7,8,9,10,11,12]. Early reporting at the initial stages of the pandemic from China, presented by several research teams, including Guan et al. (2020) [13], Zhao et al. (2020) [14]; and Mo et al. (2020) [15], had already indicated that the majority of infected patients were male, with a predisposition toward more severe cases. Despite this, global data show that the incidence of COVID-19 among males and females is both country and regionally diversified [16]. For example, findings presented by Kocher et al. (2021) from six countries, including Belgium, Canada, Denmark, Portugal, South Korea, and Switzerland, reported more cases in women by at least six percentage points [17]. Even on the small spatial scale, spatio-analyses have documented variability in the determinants of COVID-19 spread [18]. Time is an additional significant component, where dynamism and variability have been observed regarding COVID-19 gender trends throughout different periods [19]. For example, Danielsen et al. (2022) indicated that 72.7% of the difference in mortality rate between men and women was accrued in the first seven weeks of the pandemic, whereas, later, these differences were attenuated in subsequent phases. 

Proponents of the above conclusions have explored why men are more vulnerable to worse outcomes, where both biological (sex), such as weaker immune responses, and socio-cultural behavioral (gender) factors have found to be at play [20]. Bwire (2020) suggests several possible factors that explain the gender gap, including the higher expression of angiotensin-converting enzyme-2 (ACE 2; receptors for coronavirus) in males than females, and sex-based immunological differences driven by the X chromosome and gender behavior (lifestyle), such as higher levels of smoking and drinking among men. In addition, the differences in mortality between men and women have been attributed to health-seeking behavior, where findings from the United States prior to the COVID-19 outbreak have indicated that women seek health care more actively than men [21]. Findings from Spain have also indicated that females had more responsible attitudes and preventive measure behaviors (such as frequent handwashing, mask wearing and obedience regarding stay-at-home orders) than men [22].

Experience from previous outbreaks has shown the necessity of integrating a gender analysis into the efforts of preparedness and response to ensure the improved effectiveness of health interventions and promotion of gender and health equity goals. The aim of this study was to compare morbidity and mortality during two years of the COVID-19 pandemic in Israel in female and male patients with COVID-19, as well as to assess length of stay, time of health-seeking behavior after positive diagnosis, and vaccination differences. To the authors’ knowledge, this is the first study that assesses the phenomena in the Israeli COVID-19 context, and which aims to integrate the above assessments to paint a more complete COVID-19 gender analysis. 

## 2. Materials and Methods

### 2.1. Data Sources

A retrospective-archive study was conducted in Israel from 1 March 2020 to 1 March 2022 (two consecutive years). Data were obtained from the open COVID-19 database of the Israeli Ministry of Health’s (MOH), (https://data.gov.il/dataset/COVID-19 (accessed on 15 March 2022)), which encompasses information on 1270 localities and is updated daily. The database contains the number of COVID-19 diagnostic tests performed daily, confirmed cases (i.e., those that tested positive by real-time quantitative reverse-transcriptase polymerase-chain-reaction (qRT-PCR) assay—a person who tested positive was confirmed to be infected with COVID-19 regardless of the presence of any clinical symptoms and reoccurrence cases were removed in the dataset), classification of hospitalized patients, deaths by age, gender groups and vaccination status (four doses). Vaccination was documented as the number of those vaccinated in the first dose (starting from 20 December 2020), the number of those vaccinated in the second dose (as of 1 October 2021), the number of those vaccinated in the third dose (as of 30 July 2021), and the number of those vaccinated in the fourth dose (as of 2 January 2022). Hospitalized COVID-19 patients were classified according to WHO classification (mild, moderate, and severe disease, the latter classification including patients who were intubated and mechanically ventilated). Classification of hospitalized patients was conducted according to the last status received that day (for example, if a hospitalized patient was admitted in a serious condition but, at the end of the day, his/her condition improved to moderate, the patient status was defined as moderate. The above variables were selected for assessment resulting from their completeness in the online database (as they were regularly updated by the Ministry of Health). Furthermore, the majority of these variables were the point of assessment in additional manuscripts on the subject. 

### 2.2. Data Analysis

MOH data on confirmed COVID-19 cases, disease severity and deaths were analyzed by age and gender group. Status of hospitalized and severity status of COVID-19 (mildly ill, moderately ill, severely ill, critically ill and intubated patients, where patients were evaluated on the 11th day of each month) of the relevant month. The data from the Ministry of Health database became available on the 11 March 2020. Confirmed cases and deaths were calculated as a cumulative number for the relevant month. Rate of incidence cases in the population was the number of positive cases divided by the size of the relevant population group. The data were divided into five waves ((Wave 1—February–May 2020), (Wave 2—June–October 2020), (Wave 3—November 2020–March 2021), (Wave 4—April–October 2021), and (Wave 5—November 2021–March 2022)). A survival analysis was also performed for the patients who passed away (n = 10,145). The primary outcome variable was time to death, constructed as the time between date of being positive and death (failure), with censoring on 1 March 2022 for individuals who were alive by the end of the study period. We also included two secondary outcomes-time from being positive to hospital admission and in-hospital length of stay. The survival analysis included dichotomic values for gender and vaccination status (0 or 1—at least one dose). The Kaplan–Meier method was used to plot survival curves. These graphs served to test the proportional hazard assumption. We also conducted *t*-test analysis (for independent variables) to compare mean values. In addition, we conducted normality tests using the Kolmogorov-Smirnoff test. The data were analyzed using the statistical package software SPSS version 28 IBM SPSS 28.0 Statistics (IBM Corp. Released 2021. IBM SPSS Statistics for Windows, Version 28.0. IBM Corp., Armonk, NY, USA). The sample size calculation was calculated according to the guidelines of retrospective studies provided by Sackett, Haynes and Tugwell (1995) suggesting a need for 10 cases per variable [23].

## 3. Results

During the two years of the pandemic, 3,605,400 people were infected with the COVID-19 virus. Although the proportion of females (49.9%) and males (50.1%) is very similar in Israel, female infections are 1.12 times higher than males (n = 1,908,442 vs. n = 1,696,958), across almost all age groups, with the exception of the youngest (0–19) age groups. Despite this, less severe of illness and lower mortality were observed among women as compared to men, in all age groups; see Table 1. 

In addition to the higher rate of confirmed cases among women, vaccination uptake was higher among women as compared to men for all doses. The relative risk (male/female) for the first, second, third and the fourth dose were: 0.967, 0.963, 0.958 and 0.936, respectively; see Table 2. 

**Figure 1:**
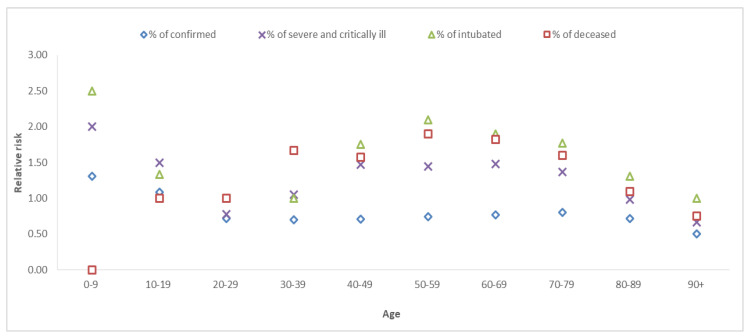
Relative risk between males and females (male/female), by age.

Corresponding to the above findings, a lower relative risk of diagnosis with COVID-19 was observed among men when compared to women (apart from the youngest (0–19) age groups); however, the relative risk of severe illness, intubation and mortality is higher among men. Furthermore, the relative risk between males and females in the youngest group (0–9) is observed to be larger, for example, in intubation, the relative risk is equal to 2.5; see Figure 1.

**Figure 2:**
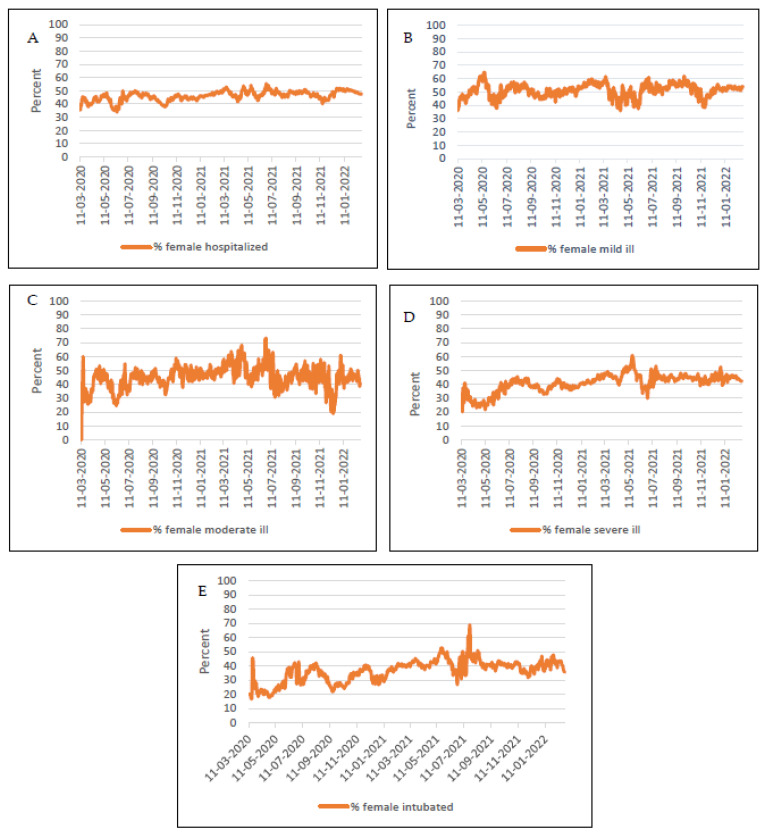
Gender trends in morbidity through five parameters including (**A**) hospitalization (out of all hospitalized), (**B**) mild, (**C**) moderate and (**D**) severe illness (among each respective population), and (**E**) intubation (among all intubated) throughout two years of the pandemic. The % males of each variable are the mirror reflection to the above data. Notes: The differences in all five trends are statistically significant according to the independent *t*-test. Statistical significance was defined as x < 0.05.

Figure 2 depicts changes over time in morbidity through five parameters, including hospitalization, mild, moderate and severe illness, and intubation for the five waves of the pandemic.

The hospitalization rate was higher among men compared to women for the entire pandemic period. These differences were higher in the beginning of pandemic (2020), but later become narrower, in 2021. 

The differences in mild and moderate disease among men and women were interchanging and irregular. These findings were different in the case of severe and intubated cases. In the beginning of the pandemic, the observed gender gaps in the percentage of severe and intubated were wider between men and women. Between the end of the third wave and start of the fourth wave, the gap was attenuated wherever an alteration between gender groups (%) is observed; see Figure 2.

**Figure 3:**
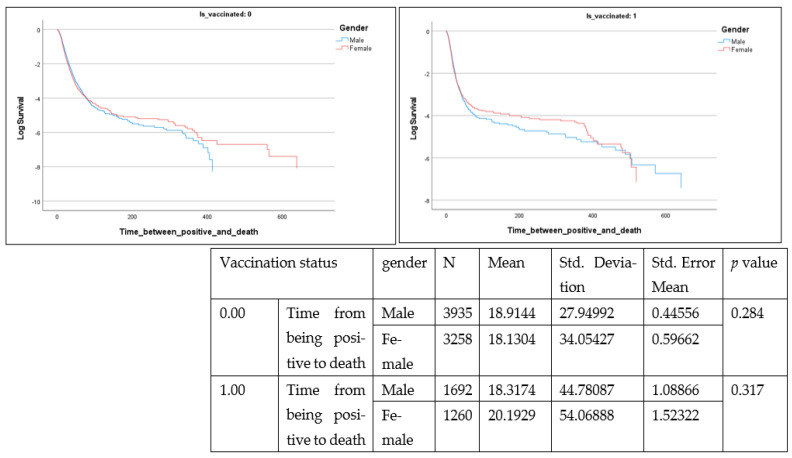
Time from positive diagnosis to death (days) (Is_vaccinated 1 = vaccinated with at least one dose).

**Figure 4:**
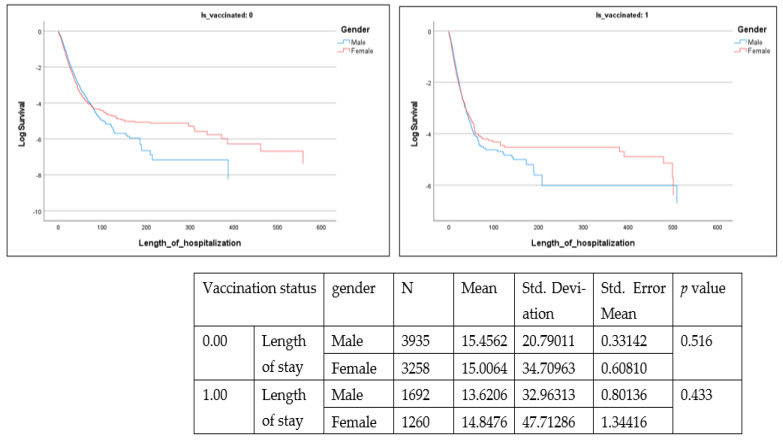
Length of stay (LOS) (Is_vaccinated 1 = vaccinated with at least one dose). = vaccinated with at least one dose).

**Figure 5:**
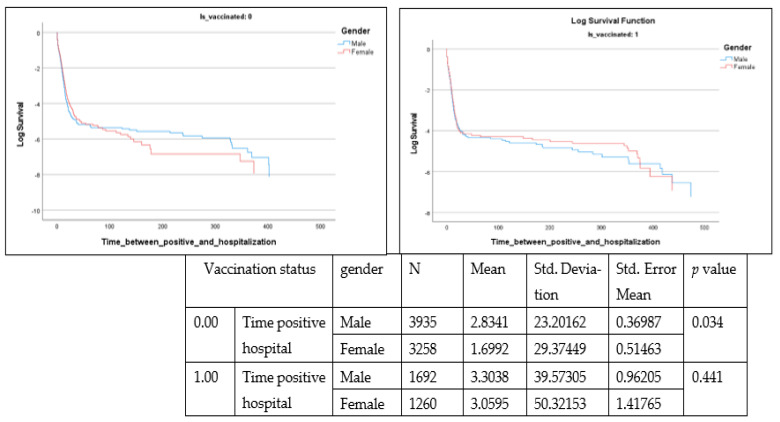
Time from positive diagnosis to hospital admittance ((Is vaccinated 1 = vaccinated with at least one dose).

Figure 3, Figure 4 and Figure 5 present survival analysis for deceased patients (n = 10,145) where three parameters are examined, including time from positive diagnosis to death, in-hospital length of stay among deceased patients and time from positive diagnosis to hospital admission. 

These parameters are presented according to vaccination status (0 or vaccinated with one dose) as well as by gender. From the data, time from positive diagnosis to death and in hospital length of stay are insignificant, whereas time from positive diagnosis to hospital admission is significant (Lon rank = 9.722; *p* = 0.002) among the non-vaccinated population. 

Among the deceased, 55.5% were men (n = 5627) and 44.5% were women (n = 4518). The mean number of days taken by unvaccinated men from positive diagnosis to hospital admission was greater (mean = 2.83, SE 23.2) than that among unvaccinated women (mean = 1.69, SE 29.4) (t = −1.830, *p* = 0.034).

## 4. Discussion

Pandemics and recessions have the potential to exacerbate health inequalities [24]. For the response to disease outbreaks such as COVID-19 to be effective and not reproduce or perpetuate gender and health inequities, it is important that gender relations that influence differential vulnerability to infection, exposure to pathogens, and treatment received, must be considered, and addressed. The findings of this study in Israel indicate that women have a higher risk of infection but are less severely ill. These findings are in line with much of the previous conclusions in the literature, that more severe illness is observed in men [7,8,9,10,11,25]; however, in contrast to previous data, we found women to have a higher incidence of viral infections [26], where Krause et al. (2020) found that women have a higher viral infection incidence. 

To explain the above gender gaps in the context of COVID-19 morbidity and mortality in Israel, several additional findings from the current study may partially explain the above phenomenon. As shown, the higher vaccination rates in women may play a protective role against the higher severity of adverse outcomes observed in men [27]. Interestingly, in contrast to our findings, a meta-analysis indicated that a majority (58%) of papers reported men as having higher intentions to get vaccinated against COVID-19 as compared to women [28]. Even findings from Israel from the beginning of the vaccination rollout campaign indicated that women were more hesitant to be vaccinated [10].

Moreover, a noteworthy component is that, despite the higher vaccination rates among women, we observe a higher incidence of infection. This may be explained by exposure risk, where women face a higher exposure to disease. For example, in terms of occupational exposure, women make up a large percentage of healthcare workers globally and may play a role as the healthcare sector faces a higher risk through interaction with patients and interaction with other healthcare workers [29].

Additionally, it has been documented that woman are more likely to have the role of primary caregivers and be involved frontline interactions with communities for essential demands [30,31]. These social commitments may be the partial exposure source for a higher risk of contagion among women as compared to males. 

In addition to these elements, our findings indicate that time from positive diagnosis to hospital admission is higher among unvaccinated men than women among those who passed away, indicating that differences in health-seeking behavior may also explain the differences in mortality, where the active seeking of care by men only occurs when the condition reaches a grave stage. Overall, the findings point to the fact that males may downplay the disease, and report for testing less in the case of slight illness, resulting in a worse aggregated picture (where more severe cases are observed among the population, while lighter ones go unidentified). The literature also has probed additional explanations for the unequal distribution of disease severity and mortality between genders, including a multifactorial phenomenon involving lifestyle differences, differences in prevalence of underlying conditions (e.g., heart disease and diabetes), a stronger immune system among women, etc. [13,32].

The above findings regarding influential factors on health-seeking behavior (hospital admission time) and vaccination differences may be indicative of a general higher risk perception among women, resulting in a higher likelihood of practicing preventive behaviors and avoidance of risk behaviors, as previously presented in findings from Spain [22,33].

Our findings indicate that health trends cannot necessarily be generalized to all countries and are very much dynamic and contingent on socio-geographical context. In addition, it is necessary to consider the specific characteristics of the disease and the various risk factors and their intersection with one another when defining an individual’s vulnerability to the impacts of COVID-19. Targeted approaches, which take into consideration sex and gender and the intersecting factors, are necessary to engage in the fight against COVID-19 to ensure the most effective and equitable pandemic response. Examples of such targeted responses, include tailored risk communication campaigns and health communication strategies which take into consideration the sex and gender component and better reflect the vulnerability of the population groups. Furthermore, further studies are needed to decipher the role of genetic, biological cultural, psychological, and environmental components that may play an important role in the varied vulnerability of the population groups.

## 5. Limitations of This Study

Several limitations of this study must be considered. The current study is based on the Open Database of the Israeli Ministry of Health, which contains aggregated data regarding vaccination and confirmation rate. In the absence of individual-level data, analyses and conclusions are purely ecological for theses variables. Despite vaccination of large proportions of the population, new variants continue, challenging the healthcare system, and contributing to a fifth and potential future waves. The findings of this study may reduce the ability to forecast the gender attributes of the “upcoming” waves. 

The analysis included repeated COVID-19 tests since some people do multiple tests before being labeled “confirmed”. Indeed, it might lead to an underestimation of the true rate of confirmed tests. However, since the national method of data collection and reporting has been stable throughout the months of the pandemic, and since, at the time of the study, COVID testing was free of charge for the entire Israeli population, we believe that this should not create a bias. Furthermore, once an individual is labeled as positive, the positive reoccurrence cases have been removed in the MOH COVID-19 database., In addition, we do not have access to data regarding hospitalization characteristics for deceased patients (e.g., complications, intubation days, ECMO use and other cardiovascular support). 

## 6. Conclusions

This study offers an important longitudinal dataset of two years to explore how sex may have played a factor in variations in COVID-19 transmission, illness, and death in Israel. The findings of this study indicate that female infections, across almost all age groups, are 1.12 times more likely (apart from the youngest (0–19) age groups). Despite this, the relative risk of severe illness, intubation and mortality is higher among men throughout the two years of the pandemic. In addition, our findings indicate that the mean number of days taken by unvaccinated men from positive diagnosis to hospital admission was greater than among unvaccinated women among the deceased population, potentially indicating that differences in health-seeking behavior may also contribute to differences in mortality in this context. Going forward, the results of this study indicate that targeted approaches, which take into consideration sex and gender and the intersecting factors, are necessary to engage in the fight against COVID-19 to ensure the most effective and equitable pandemic response. Furthermore, additional studies are needed in the future to elucidate the role of genetic, biological, cultural, psychological, and environmental components that may play an important role in varied vulnerability of differing population groups. A better understanding of the assorted factors are necessary to enhance the strategies that are utilized to promote preventive behavior, alongside adapting treatment protocols and therapies to respond to the differences in disease course. 

## Figures and Tables

**Table 1:** Descriptive statistics of COVID-19 cases from 1 March 20 until 1 March 22, (n = 3,605,400).

Age Group	Gender	Num. of Testes	Num. of Confirmed Cases	% of Confirmed Cases among Individuals Tested	Num. of Hospitalized	% of Hospitalized among Those Confirmed Cases	Num. of Deceased Cases	% of Deceased Cases among Those Confirmed Cases	Num. of Intubated Cases	% of Intubated Cases among Those Confirmed Cases	Num. of Severe and Critically Ill	% of Severe and Critically Ill among Those Confirmed Cases
0–9	Male	3,604,318	396,475	11	2771	0.699	7	0.002	28	0.007	100	0.025
0–9	Female	3,614,679	303,633	8.4	2254	0.742	4	0.001	13	0.004	63	0.021
10–19	Male	3,612,414	400,978	11.1	965	0.241	7	0.002	23	0.006	70	0.017
10–19	Female	3,592,314	366,416	10.2	1090	0.297	7	0.002	20	0.005	62	0.017
20–29	Male	3,607,403	223,659	6.2	1524	0.681	24	0.011	49	0.022	207	0.093
20–29	Female	3,584,826	308,295	8.6	4861	1.577	21	0.007	54	0.018	247	0.080
30–39	Male	3,585,475	211,543	5.9	2163	1.022	50	0.024	94	0.044	568	0.269
30–39	Female	3,614,929	303,654	8.4	4937	1.626	35	0.012	92	0.030	546	0.180
40–49	Male	3,632,673	188,899	5.2	3469	1.836	115	0.061	241	0.128	1308	0.692
40–49	Female	3,617,795	264,099	7.3	3444	1.304	74	0.028	138	0.052	896	0.339
50–59	Male	3,567,629	124,867	3.5	4735	3.792	366	0.293	518	0.415	2160	1.730
50–59	Female	3,612,021	169,765	4.7	3772	2.222	191	0.113	249	0.147	1493	0.879
60–69	Male	3,653,652	84,034	2.3	6319	7.520	947	1.127	885	1.053	3139	3.735
60–69	Female	3,576,867	107,306	3	4646	4.330	524	0.488	467	0.435	2119	1.975
70–79	Male	3,735,333	44,824	1.2	6714	14.979	1550	3.458	993	2.215	3603	8.038
70–79	Female	3,512,667	52,690	1.5	5483	10.406	975	1.850	560	1.063	2626	4.984
80–89	Male	3,516,000	17,580	0.5	5439	30.939	1795	10.210	613	3.487	2943	16.741
80–89	Female	3,518,286	24,628	0.7	5949	24.155	1647	6.688	464	1.884	3006	12.206
90+	Male	4,099,000	4099	0.1	1796	43.816	803	19.590	130	3.172	990	24.152
90+	Female	3,978,000	7956	0.2	2700	33.937	1070	13.449	130	1.634	1479	18.590

**Table 2:** Differences in vaccination uptake between males and females.

Gender	First Dose	Second Dose	Third Dose	Fourth Dose
**Male**	3,273,901	2,881,961	2,166,722	351,205
**Female**	3,372,618	2,979,819	2,252,895	373,638
**Vaccinated per total male population**	0.68198	0.599647	0.470637	0.078054
**Vaccinated per total female population**	0.704551	0.622494	0.470637	0.078054
**Relative risk** **(Fraction of vaccinated males/Fraction of vaccinated females)**	0.967	0.963	0.958	0.936

## Data Availability

Links to publicly archived dataset: https://datadashboard.health.gov.il/COVID-19/general (accessed on 15 March 2022).
